# Biomarker of Stress, Metabolic Syndrome and Human Health

**DOI:** 10.3390/nu14142935

**Published:** 2022-07-18

**Authors:** Jean-Baptiste Bouillon-Minois, Frédéric Dutheil

**Affiliations:** 1 Université Clermont Auvergne, CNRS, LaPSCo, Physiological and Psychosocial Stress, F-63000 Clermont-Ferrand, France; fdutheil@chu-clermontferrand.fr; 2 CHU Clermont-Ferrand, Emergency Medicine, F-63000 Clermont-Ferrand, France; 3 CHU Clermont-Ferrand, Occupational and Environmental Medicine, WittyFit, F-63000 Clermont-Ferrand, France

Metabolic syndrome is a significant public health concern linked to the obesity pandemic. Even if glycemia, triglycerides, and HDL are mandatory for the assessment of metabolic syndrome, other biomarkers have recently been proposed to be linked. Recent studies indicate that both chronic and (repeated) acute stress are involved in developing metabolic syndrome. Furthermore, both oxidative and psychosocial stress have been linked to heart disease and metabolic syndrome. The main hypothesis is the disruption of the hypothalamic–pituitary–adrenal axis (HPA axis). Indeed, a dysfunction in the HPA axis increases cortisol levels in the blood, increasing both glucose and insulin levels, causing the apparition of insulin resistance, the promotion of dyslipidemia, high blood pressure, and visceral adiposity. Secondly, HPA axis dysfunction has an impact on bones, cardiovascular diseases, and psychiatric disorders.

This Special Issue is composed of five articles, three original articles and two reviews, including a systematic review with a meta-analysis. 

The first study concerns 535 obese patients aged 0–18 years. Children born large for gestational age predominated over those born small for gestational age. Birth weight had an independent effect on triglycerides and insulin resistance (two well-known biomarkers of cardiometabolic risk) during childhood, whereas obesity had a direct influence on hypertension, an impaired glucose metabolism, and hypertriglyceridemia [1].

The second one also studied pediatric obesity, but this time, the impact of acute stress was assessed for 137 obese youngsters with the Trier Social Stress Test. Those overweight and with a high level of chronic stress seemed to have a higher stress vulnerability (stronger relative salivary cortisol reactivity and weaker happiness recovery) and a higher fat/sweet snack intake. Those patients would benefit from stress therapy to reduce the risk of obesity [2].

The third one studied the link between dysfunction pancreatic ß-cells and nonalcoholic fatty liver disease (NAFLD). This disease is associated with a decreased insulin sensitivity. Among 6168 participants, those with NAFLD had a much higher HOMA2-%B level. However, when evaluating the β-cell function in the context of insulin resistance by using the disposition index, NAFLD subjects had a lower disposition index. Thus, it seems that pancreatic β-cell function might be a novel predictor for the presence of NAFLD, and an insufficient compensatory β-cell function is associated with NAFLD [3].

The fourth one explored the link between metabolic syndrome and sarcopenia—two common ailments among elderly patients. Indeed, skeletal muscle is a major organ in the glucose metabolism. The loss of muscle mass has been closely linked to insulin resistance and metabolic syndrome through the accumulation of intramuscular fat using a combination of factors (oxidative stress, inflammatory cytokines, mitochondrial dysfunction, insulin resistance, and inactivity). Persistent inflammation, fat deposition, and insulin resistance are thought to play a complex role in the association between metabolic syndrome and sarcopenia, both affecting quality of life and contributing to increased frailty, weakness, dependence, morbidity, and mortality [4].

Lastly, leptin, the main satiety hormone presenting a circadian rhythm, was studied in an acute way. Indeed, it seems that leptin can be considered a biomarker of acute stress, with a 34% decrease following acute stress. Individuals with a normal weight and women had a higher variation of leptin levels after stress, suggesting that leptin may have implications in obesity development in response to stress in a sex-dependent manner [5].

In conclusion, it was an honor to be a guest editor to this very interesting Special Issue. All the different findings provide a higher comprehension on the link between metabolic syndrome and stress through the use of novel biomarkers. We hope that future research is performed, aiming to find novel pathway to increase quality of life among obese and stressed people.
